# The role and mechanism of 5-HTDRN-BNST neural circuit in anxiety and fear lesions

**DOI:** 10.3389/fnins.2024.1362899

**Published:** 2024-05-09

**Authors:** Xianli Zheng, Li Dingpeng, Xingke Yan, Xiaoqiang Yao, Yongrui Wang

**Affiliations:** ^1^Gansu University of Traditional Chinese Medicine, Lanzhou, Gansu, China; ^2^Affiliated Hospital of Gansu University of Traditional Chinese Medicine, Lanzhou, Gansu, China; ^3^Gansu Provincial Second People’s Hospital, Lanzhou, Gansu, China

**Keywords:** 5-HTDRN-BNST neural circuit, anxiety, fear, mechanism progress, stress, dysfunction

## Abstract

Central 5-hydroxytryptaminergic dorsal raphe nucleus (5-HTDRN)-bed nucleus of stria terminalis (BNST) neural circuit dysfunction is one of the important neurobiological basis of anxiety and fear disorders. Under stress, 5-hydroxytryptamine (5-HT) neurons act on BNST receptors to attenuate anxiety and fear responses or enhance anxiety and fear. In BNST, corticotropin releasing factor neurons play a role in regulating emotions by reversely regulating excitatory or inhibitory 5-HT neurons. The composition of 5-HTDRN-BNST neural circuit, the pathological changes of 5-HTDRN-BNST neural circuit function damage under stress, and the effects of 5-HTDRN-BNST neural circuit on anxiety disorder, panic disorder and post-traumatic stress disorder were analyzed and are summarized in this paper. The characteristics of functional changes of the neural circuit and its effects on brain functional activities provide a basis and ideas for the treatment of anxiety and fear disorders through the regulation of 5-HTDRN-BNST neural circuit, and they also provide a new perspective for understanding the pathological mechanism of such diseases.

## 1 Introduction

When the body is transiently or continuously exposed to trauma and acute stress, people will experience a variety of negative emotions such as fear, anxiety, and depression. Most of these risk factors are classified as exposure to traumatic events. These emotions are mainly due to the central nervous system affecting the neurotransmitter system and neural circuits, thus affecting the neuroendocrine system, and ultimately affecting emotional behavior. 5-hydroxytryptamine (5-HT) is a monoamine neurotransmitter, which exists in the central nervous system and participates in a series of behaviors, personality and emotional activities of the human body. Therefore, the latest research has found that the treatment of these diseases usually focuses on the regulation of 5-HT system (Michopoulos et al., 2017). Anxiety and fear are the basic pathogenic emotions in traumatic and stressful events. They can activate the 5-HT neuron system, and the 5-HT released is one of the neurotransmitters that regulate the input of damaging signals. It is involved in regulating behavioral and physiological processes, including emotions (anxiety, depression, fear), feeding, reward, learning and memory, and aggressive behavior, affecting the brain activity of its projected brain regions, causing changes in central and peripheral neurotransmitters and receptors, and finally reversely regulating anxiety and fear emotional behaviors and processes (Shuang et al., 2018; Qiang and Lin, 2020). Half of the 5-HT neurons are mainly concentrated in the dorsal raphe nucleus (DRN), a heterogeneous brain region, and other neurons present in the DRN regulate the activity of local 5-HT neurons. Therefore, the 5-HT subtype is polymorphic. The main brain area projected by 5-HT neurons is the bed nucleus of the stria terminalis (BNST) on the ventral side of the forebrain. It is one of the main target brain areas of the DRN’s 5-HT neural projection circuit and contains various subtypes of 5-HT receptors. The BNST is in a key position in the stress response of the neural circuit and can participate in the expression and regulation of organism-related emotions (such as fear and anxiety) by connecting the output of the external stimulus information and feedback behavior (Avery et al., 2016). The 5-HT neurons in the DRN release neurotransmitters and interact with various subtypes of receptors, activating other neurons and forming conflict systems such as anxiety, depression, and fear. These complex neural circuits are interconnected, among which the 5-HTDRN-BNST neural circuit is involved in inducing behavioral responses similar to anxiety and fear (Paul et al., 2014).

Under the DRN and various neurons and subtypes, 5-HT forms a conflict system of anxiety, depression, and fear, which is interconnected to form a complex neural circuit. It is involved in inducing anxiety-like and fear-like behavioral responses (Paul et al., 2014). At present, there are only a few studies and reviews on the 5-HTDRN-BNST neural circuit in China. Therefore, this article summarizes relevant foreign research, reviews the literature, and explores the mechanism of 5-HTDRN-BNST loop regulation and the regulation of anxiety, fear, and panic in stress and traumatic events from the aspects of its mechanism, pathological changes, and the loop mechanism for anxiety, fear, and panic. The aim is to provide a theoretical basis for clinical treatment.

## 2 The physiological mechanism of the 1 5-HTDRN-BNST loop

5-HT neurons have a wide range of nerve fiber projections to the DRN and BNST. This forms a complex neural circuit with various parts of the brain region and participates in emotional and emotional regulation. The specific function of the 5-HTDRN-BNST neural circuit remains unknown, and how this circuit participates in diseases with symptoms of fear and anxiety such as anxiety disorders, panic disorder, and post-traumatic stress disorder are rarely reported. Additionally, the specific mechanism is not clear. The changes in the functional activity of the 5-HTDRN-BNST circuit affect the regulation of anxiety and fear emotions in the body. The main mechanism is that 5-HT neurons release the neurotransmitter 5-HT, which acts on BNST brain receptors to weaken stress-induced anxiety and fear responses or enhance anxiety and fear emotions (Pelrine et al., 2016; Hammack et al., 2021). Under stress, corticotropin releasing factor (CRF) neurons in BNST release CRF, which exerts an emotional regulatory effect by reverse regulating the excitation or inhibition of 5-HT neurons by interacting with receptors. Neurons in DRN and BNST brain regions project to each other to produce information interaction, respond to and judge external stimulus events, and transmit information downstream to make instinctive defense behavior (McDevitt et al., 2014; Marcinkiewcz et al., 2016). The neural circuit of the human brain is involved in various emotional cognition and behaviors. 5-HT neurons establish interconnections through collective behavior, integrate neural signaling through excitatory and inhibitory activities, and form the 5-HTDRN-BNST loop to perform various functions (Xinghui et al., 2021). Therefore, anxiety and fear-based disease characteristics are often accompanied by abnormal changes in the 5-HTDRN-BNST loop, which is one of the important neuropsychological bases leading to the occurrence of such diseases.

## 3 The pathological mechanism of the 2 5-HTDRN-BNST loop

### 3.1 Activation of 5-HT neuronal function in DRN-BNST

The emotional regulation of 5-HT neurons is mainly due to neuronal hyperpolarization and inhibition of presynaptic and postsynaptic BNST neuronal neurotransmitter and receptor mechanisms in the presynaptic and postsynaptic BNST brain regions to weaken stress-induced anxiety responses. In DRN, 5-HT neurons directly project to BNST, integrate internal information and post-output, and closely cooperate with the medial prefrontal cortex (mPFC) to control anxiety and fear (Linley et al., 2017; Glover et al., 2020). 5-HT neurons regulate the activity of multiple neurons in BNST, improve anxiety-like behavior, and involve various 5-HT neurotransmitters and receptor subtypes (5-HT1A, 5-HT2C, etc.) as well as relative receptor expression patterns in normal and pathological anxiety states (Guo et al., 2009). Stress stimulates DRN to release 5-HT neurotransmitters, and stimulates the expression of postsynaptic 5-HT1A receptors in BNST brain regions, which have anti-anxiety effects. Stimulation of postsynaptic 5-HT2C receptors in BNST enhances anxiety and reverse learning. The 5-HT neurotransmitters released from DRN can also play a role in stimulating 5-HT neurons in DRN. The excitation of the presynaptic 5-HT1A heteroreceptor can exert anti-anxiety effects by inhibiting the release of glutamate from presynaptic terminals, thereby reducing the signal transduction of the postsynaptic 5-HT2C receptor (Marcinkiewcz et al., 2016). Excitation of the 5-HT1A receptor leads to the inhibition of N-type calcium channels and reduces the activity of calcium-dependent adenylate cyclase, thereby reducing the formation of cyclic adenosine monophosphate (cAMP) and lowering the activity of protein kinase A (PKA) (Meadows et al., 2017). Therefore, projecting 5-HT neurons from the DRN to the BNST activates 5-HT1AR in the BNST, which has an anti-anxiety effect. Exciting 5-HT2CR in the BNST enhances anxiety and reverse learning and has the opposite effect of synthesizing 5-HT1A receptors post synapse (Zhou et al., 2019). The study also found that optogenetics specifically activates the 5-HT neurons in the DRN-BNST pathway of anxiety model mice, which can act on BNST neurons expressing 5-HT2C receptors, resulting in anxiety symptoms (Marcinkiewcz et al., 2016). On the contrary, the activation of 5-HT released by the DRN-BNST pathway in a highly anxious environment will cause hyperpolarization of BNST neurons, but this inhibitory effect can be blocked by 5-HT1A receptor antagonists (Garcia-Garcia et al., 2017), indicating that the activation of BNST neurons expressing 5-HT1A receptors has an anti-anxiety effect. Therefore, the projection of 5-HT neurons from DRN to BNST is generally considered to enhance anxiety and reversal learning by activating the 5-HT2C receptor signal in BNST, while the 5-HT1A receptor in BNST plays the opposite role. When stimulated by stressors, the 5-HT neuronal system of the DRN-BNST circuit is excited, which affects the neuronal activity of the 5-HT neurons projecting to BNST in the brain, causing expression changes in central nervous system neurotransmitters (including 5-HT), CRF, and receptors, leading to changes in the circuit information cascade response, regulating anxiety and fear behavior and processes (Daniel and Rainnie, 2016).

### 3.2 DRN-BNST reverse regulatory factor

The 5-HT neurons in the DRN are also regulated by CRF in BNST, which can excite and inhibit the 5-HT neurons, resulting in increased 5-HT release in BNST and other marginal structures and the feedback mechanism to alleviate the stress discomfort effect.

**Figure 1:**
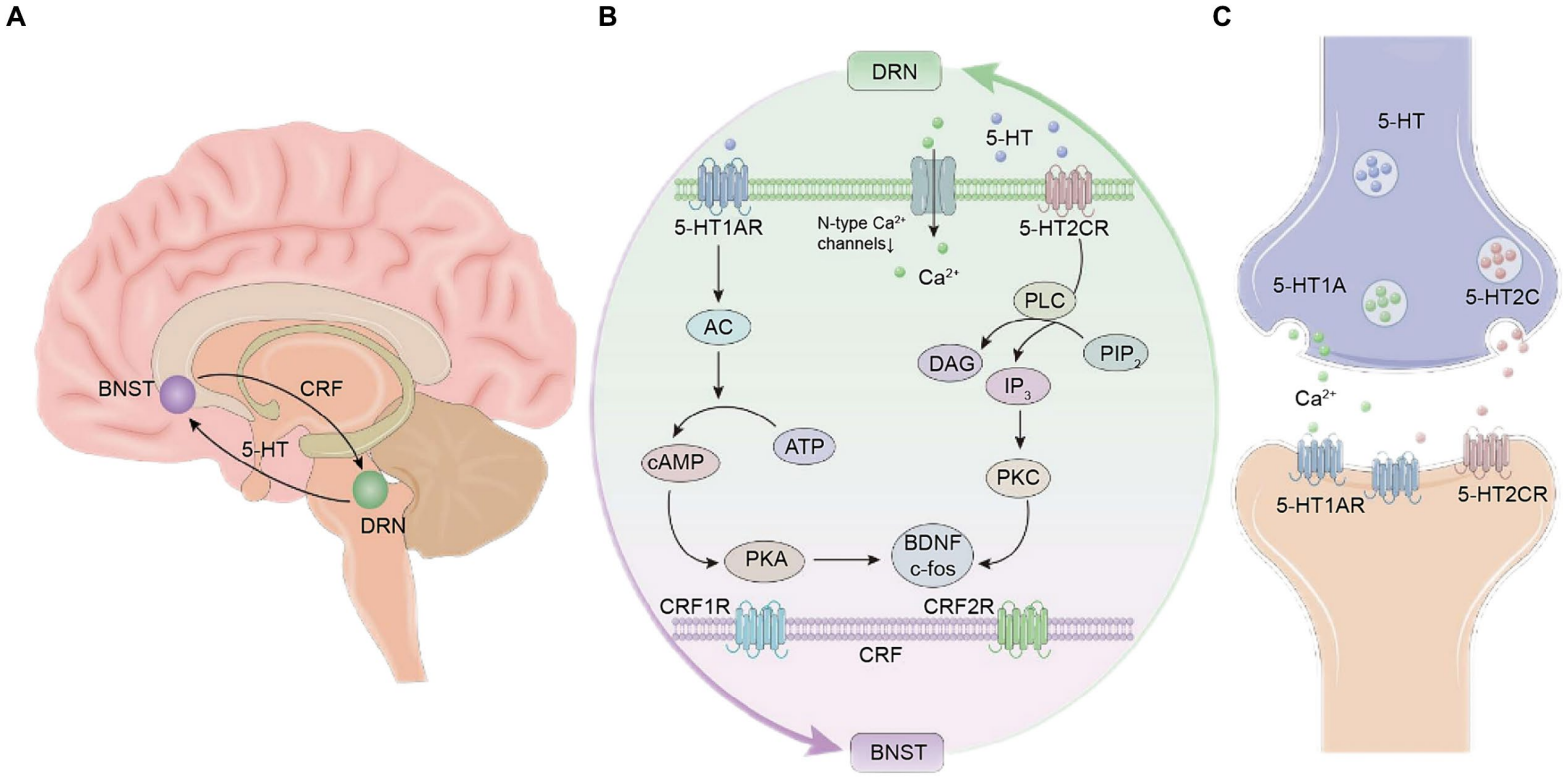
Schematic diagram of the neural circuit mechanism between the dorsal raphe nucleus (DRN) and the bed nucleus of the stria terminalis (BNST) of 5-hydroxytryptamine (5-HT). **(A)** Location of the DRN and BNST in the brain and 5-HT and corticotropin-releasing factor (CRF) neurons. **(B)** Schematic diagram of the chemical signal transduction mechanism in the neural circuits. **(C)** Schematic diagram of the mechanism of action between 5-HT neurotransmitters and receptors.

When the brain is stimulated by stress, the 5-HT neurons in DRN release neurotransmitters that enhance fear and anxiety, further activating the subfamily of CRF neurons in BNST. DRN neurons can express various CRF receptors, with CRFl receptors dominating at low doses of CRF and inhibiting the activity of 5-HT neurons in DRN when CRF1 receptors are activated. Conversely, at high doses of CRF, CRF2 receptors dominate, and 5-HT neurons in DRN are activated when CRF2 receptors are activated. The two receptors dynamically regulate the homeostatic balance of 5-HT neurons in DRN through mutual influence and antagonism. In addition, 5-HT in DRN projects to BNST through the action of 5-HT2C receptors, binds to the CRF inhibitory pathway in BNST, and inhibits the anti-anxiety and fear effects (Tiantian et al., 2010). Studies have shown that overexpression of CRH in BNST using lentiviral vectors can regulate conditioned anxiety (i.e., persistent fear enhanced startle). These behavioral changes may be due to compensatory changes that lead to decreased expression of CRHR1 receptor in BNST and decreased expression of CRHR2 receptors in DRN (Rajbhandari and Bakshi, 2020). In addition, during stress stimulation, 5-HT in DRN enhances fear and anxiety, and activates the central CRF neurons in the BNST of mice to release CRF. BNST is rich in CRF and norepinephrine (NE) receptors, further promoting the release of NE and CRF in the brain, leading to increased sensitivity of NE receptors in the basal lateral amygdala (BLA). The increased release of NE may alter the activity of CRF receptors in BNST to regulate the startle response index of interrupted pre pulse suppression (Garakani et al., 2020; Figure 1).

## 4 The regulatory mechanism of 5-HTDRN-BNST loop in mood disorder diseases

Mental disorders based on anxiety and fear are the most common emotional health disorders, which are manifested in anxiety disorders, panic disorder, post-traumatic stress disorder and other diseases. They have many similarities in symptoms, treatment, and etiology. The impaired function of the 5-HTDRN-BNST circuit under stress leads to anxiety and fear symptoms, which may reflect that these diseases share similar symptoms and/or have a similar biological basis, affect brain neural network activity, and change neurotransmitters and receptors to regulate emotional behavior and fear processes (Singewald et al., 2015). The 5-HTDRN-BNST circuit plays an important role in the pathogenesis and treatment of anxiety and fear diseases and will provide new regional targets for the treatment of such diseases.

### 4.1 Mechanism of the 5-HTDRN-BNST loop in regulating anxiety disorder

Anxiety disorder (AD) is a mental disorder of brain dysfunction characterized by persistent anxiety, fear, tension, and autonomic nervous activity disorders (Marcinkiewcz et al., 2019). Cannabidiol (CBD) in BNST can reduce the expression of environmental fear conditioning and anxiety-like behavior. It can be used as a 5-HT1A agonist to enhance the increased heart rate during restraint stress through the 5-HT1A receptor in BNST, which is consistent with the effect of 5-HT1A activation on inhibiting BNST to activate the parasympathetic nervous system to regulate the heart rate during acute stress (Crestani et al., 2013). 5-HT in DRN plays a pre-synthesis role in BNST and regulates glutamatergic transmission. The release of 5-HT in BNST after stress may counteract the promotion of glutamatergic transmission to BNST caused by CRF (Guo and Rainnie, 2010). Activation of the 5-HT1B receptor in BNST may limit the release of other transmitters into BNST, thus providing inhibitory control of the post-stress anxiety response (Tiantian et al., 2010). All cell types located in BNST in the 5-HTDRN-BNST neural circuit express the 5-HT1A receptor mRNA. 5-HT in DRN can act on the 5-HT1A receptor in BNST to cause a hyperpolarization inhibition reaction, resulting in a decrease in anxiety-like behavior. It can also act on the 5-HT2C receptor in CRF neurons in BNST to further enhance its activity, depolarize neurons, and promote anxiety-like behavior (Tiantian et al., 2010). The ability of 5-HT to inhibit BNST activity by acting on 5-HT1A and 5-HT1B receptors weakens the pre-feedback-increased activity of 5-HT2C. Therefore, the effect of 5-HT on anxiety-like behavior may be heavily dependent on the balance of excitatory and inhibitory 5-HT receptors in BNST (Zhou et al., 2019). In BNST, CRF activates 5-HT neurons in DRN to form a negative feedback loop, which reduces anxiety levels in the presence of stressors (Hammack et al., 2009). 5-HT in DRN is projected to BNST through the action of 5-HT2C receptor and binds to the CRF inhibitory microcircuit in BNST, which silences the anti-anxiety BNST output to the ventral tegmental area (VTA) and lateral hypothalamus (LH). Therefore, the DRN5-HT-BNSTCRF loop regulates fear and anxiety, and the negative feedback regulation of the loop can explain the occurrence of adverse events in patients with anxiety disorders in the early treatment of selective serotonin reuptake inhibitors (SSRIs) (Marcinkiewcz et al., 2016). Therefore, under stress, CRF activates 5-HT neurons in DRN, and the effect of 5-HT on BNST mainly plays an inhibitory role, thereby inhibiting the further release of CRF in BNST and reducing the anxiety response. Maintaining the balance of 5-HT receptor subtypes in BNST can support this negative feedback loop, which is conducive to maintaining the state of inhibition. The above studies demonstrate that the 5-HTDRN-BNST neural circuit bidirectionally regulates anxiety and plays an indispensable role in the pathogenesis of anxiety disorders.

### 4.2 Mechanism of the 5-HTDRN-BNST loop regulating panic disorder

The disorder of emotional processing in patients with panic disorder (PD) is related to the abnormal activation of DRN and BNST in the midbrain and the abnormal function of related cortical-peripheral neural circuits (Shin et al., 2020; Barnes et al., 2021). The abnormal 5-HTDRN-BNST neural circuit is also an important neuropsychological mechanism of PD emotional regulation and panic attack. BNST is involved in the regulation of long-term fear responses similar to anxiety (persistent fear) and promotes fear learning (Goode and Maren, 2017). Repeated stress exposure leads to long-term facilitation of selective synaptic plasticity in CRF neurons in the oval nucleus of the synthetic stress hormone (BNSTov) of BNST, which promotes sustained changes in stress-induced behavior, including enhanced startle reflex and fear conditioning (Dabrowska et al., 2013). This change in synaptic plasticity of CRF neurons may lead to the formation of long-term memory in the fear circuit. CRF in BNST acts on the CRF1 receptor in the DRN and regulates anxiety and negative emotions after long-term threat stimulation or detoxification (Dabrowska et al., 2016). High levels of CRF in DRN mimic the effect of uncontrollable stress by activating CRF2 receptors. Studies demonstrate that 5-HT neurons in DRN are activated during stress, and 5-HT1A receptors can inhibit the activity of aversive memory circuits in BNST. CBD in BNST attenuates the expression of c-fos in BNST after situational fear evokes 5-HT1A receptor-dependent neurons (Lemos et al., 2010). When 5-HT is released in BNST, the 5-HT1A receptor may have a significant effect on the resting membrane potential and neuronal excitability only in the presence of disgust stimuli. Knockdown of the 5-HT1A receptor aggravates the increase of neuronal excitability in contextual fear memory. The 5-HT1A receptor may have a buffering effect on the resting electric potential (RMP) after the fear condition is revealed after 5-HT1A knockdown (Marcinkiewcz et al., 2019). When the release of 5-HT in BNST is relatively low, the knockdown effect of 5-HT1A may not appear under low stress conditions, and it is also possible that the postsynaptic 5-HT1A receptor is expressed in the anxiety and anti-anxiety circuits of BNST at the same time, and their removal has a net neutral effect on anxiety-like behavior (Mazzone et al., 2018). Notably, this difference between anxiety and fear suggests that a high arousal state may induce plasticity in the 5-HT1A receptor signal, allowing the 5-HT1A receptor to act as a molecular brake to buffer overstimulation (Brinkmann et al., 2018). Experimental studies demonstrate that in light-stimulated SertCre: ChR2DRN → BNST mice, cue and environmental fear memories are significantly enhanced (Marcinkiewcz et al., 2016), BNST extends to DRN fibers, and the overexpression of CRF in the dorsolateral BNST significantly down-regulates expression of the CRF2 receptor in rat DRN (Sink et al., 2013). Thus, the 5-HT neurons in DRN are activated during stress, and the plasticity of the 5-HT1A receptor signal in BNST is induced under high arousal states, which acts as a molecular brake to regulate buffer overstimulation, while CRF in BNST acts on CRF receptors in DRN to enhance and consolidate fear memory. An abnormal 5-HTDRN-BNST neural circuit is the key mechanism in the occurrence of panic disorder.

### 4.3 Mechanism of the 5-HTDRN-BNST loop regulating post-traumatic stress disorder

Post-traumatic stress disorder (PTSD) is characterized by impaired re-experience, avoidance, negative emotional and memory processing, and can continue to develop after traumatic events (Sin et al., 2017). Studies demonstrate that fear and anxiety disorders in PTSD patients are closely related to changes in the structure and function of the central nervous system. Its complex plasticity is associated with impaired activity in the mPFC, impaired DRN function, a hyperactive BNST, and impaired fear elimination (Sengupta and Holmes, 2019; Awasthi et al., 2020). When stimulated by stressors, the stress response system is activated, and the important DRN-BNST neural circuit of fear and anxiety behavior undergoes neurochemical and morphological changes (Miles and Maren, 2019), causing the mental health disease PTSD (Maren and Holmes, 2016). The abnormal level of 5-HT reduces its effect on the inhibition of mental behavior and drives PTSD patients toward a state of continuous fear, irritability, and excitement (Lo et al., 2011). The release of 5-HT by BNST can inhibit and stimulate the activity of BNST neurons. Regulating the balance of BNST receptor subtypes to excitement may produce anxiety or fear-like phenotypes. The response of BNST to 5-HT may be mediated by a decrease in excitatory 5-HT2C receptor function to inhibit the behavioral phenotype that may mediate anti-stress (Zhou et al., 2019). In BNST, 5-HT inhibits neurons through postsynaptic and presynaptic mechanisms and can also play a role in activating 5-HT DRN neurons. The activation of the postsynaptic 5-HT1A receptor may express the anti-aversion effect of 5-HT, while activation of the presynaptic 5-HT1A heterogeneous receptor may play an anti-anxiety role by inhibiting the release of glutamate from presynaptic terminals, thereby reducing the signal transduction of the postsynaptic 5-HT2C receptor (Hammack et al., 2012). In PTSD-related anxiety and fear expression, 5-HT and CRF receptors are activated to regulate stressors. CRF afferents from BNST regulate the activity of 5-HT neurons in DRN. DRN neurons can express a variety of CRF receptors. CRF inhibits 5-HT activity in DRN by activating CRF type 1 receptors. CRF may stimulate the activity of 5-HT neurons in DRN by activating CRF type 2 receptors (Sasaki Russell et al., 2020). The study also found that mice with repeated exposure to a series of traumatic electric shocks showing PTSD-like phenotypes had long-term and continuous up-regulation of CRF2R mRNA in BNST, while CRF2R gene knockout in the posteromedial BNST prevented the development of PTSD-like features, which confirmed the bidirectional nature of CRF1R and CRF2R responses in anxiety and pain perception models.

The response of PTSD patients to unpredictable aversive stimuli is signal allergy and persistent anxiety, including periodic fear and a prolonged anxiety response (Brinkmann et al., 2017). DRN and BNST brain regions are involved in the potential dynamic stress response mechanism, which can reflect the complex emotional state associated with PTSD. Studies have shown that increasing the activity of 5-HT1AR inhibitory autoreceptors in DRN changes the 5-HT1AR regulation of GABAergic interneurons in the DRN that simultaneously express CRF2R and 5-HT1AR, or is due to the inhibition of postsynaptic receptors including 5-HT1AR and 5-HT2CR in the forebrain BNST. Changes in the activity of non-antagonistic CRF1R in the DRN of CRF2R knockout mice may also be an important factor mediating these effects or directly inhibiting the activity of 5-HT neurons (Issler et al., 2014).

In addition, experimental studies have shown that there is no significant difference in the expression of 5-HTR in DRN of CRFR2-null mice, so these effects may also be mediated by receptor reduction and desensitization (Rozeske et al., 2011). Stress activates 5-HT neurons in the DRN, which is related to the functional desensitization of 5-HT1AR. In the extrapyramidal brain region that receives DRN projection but lacks its own 5-HT1AR, the response to 5-HT1AR agonists is also weakened, indicating that CRF2R may be necessary to maintain normal basal neuronal activity in the DRN, especially to maintain the balance of 5-HT1AR function. Excessively active BNST in PTSD patients can lead to hypothalamic–pituitary–adrenal (HPA) axis dysfunction. BNST regulates the HPA axis via the hypothalamus through excitatory and inhibitory projections and may prevent the development of post-traumatic behavioral changes (Henckens, 2017). Therefore, the 5-HTDRN-BNST neural circuit plays an important regulatory role in the complex emotional state of PTSD, which may promote the stress response or regulate the protective dysfunction in the stress response, which provides a new perspective for us to further study the mechanism of abnormal emotional regulation of PTSD.

## 5 Summary

In summary, the DRN is interconnected with the BNST. As part of the extended amygdala, BNST serves as a conduit between the limbic structure of the forebrain and the hypothalamus and brainstem regions. BNST is a key location in the brain that transmits autonomic, sensory, and stress-related information to the emotional processing center of the brain. In DRN, 5-HT neurons release excess 5-HT to the BNST under inevitable stress and enhances the involvement of the basal ganglia in fear memory acquisition and the association between threatening stimuli and danger (Worley et al., 2018). BNST sends a large amount of CRF and glutamatergic inputs to regulate DRN. These structures mutually input excitatory or inhibitory signals and weaken anxiety and fear resolution by enhancing the activity differences that mPFC must overcome to control the activity of the BNST. Therefore, by activating the 5-HTDRN-BNST neural circuit, 5-HT and its projection to DRN can be dynamically regulated in both directions, which in turn affects the transmission of neural information and the integration and transduction of neural signals, regulates the function of the brain’s neuroendocrine system, and ultimately affects the emotional behavior of anxiety, fear, shock, tension, despair, and depression. In recent years, some studies have intervened and explored the mechanism of the 5-HTDRN-BNST neural circuit, preliminarily clarifying the abnormal changes in the function of the 5-HTDRN-BNST neural circuit and its impact on brain function in diseases based on anxiety, fear, and panic symptoms. The deeper mechanisms and pathways of action need to be further explored by studying the regulation of the protective function in the stress response or promoting the stress response to exert its effects. Likely, dysfunction of the 5-HTDRN-BNST neural circuit is the basis for increasing pathological anxiety, fear, and panic related diseases to the 5-HT state. Regulating the functional activity of the 5-HTDRN-BNST neural circuit can serve as a potential protective mechanism for regulating anxiety and fear emotions in the body, which involves the integration and regulation of multiple signaling pathways. In the treatment of emotional disorders such as anxiety, fear, and panic, reconstructing appropriate 5-HTDRN-BNST signals to enhance the ability of anxiety, fear, and panic resolution, changing the signal and function of the DRN-BNST neural circuit, and stimulating mPFC to exert appropriate inhibitory control on this signal is a key step in treating diseases with pathological attributes such as anxiety, fear, and panic. SSRIs are currently the first-line drugs for treating this type of disease, but they may rapidly worsen symptoms and lead to poor treatment compliance. Therefore, further research on the functional impairment of the 5-HTDRN-BNST circuit can help to understand the physiological and emotional behavioral processes of functional specificity and clarify that functional impairment of the 5-HTDRN-BNST circuit is an important factor leading to human mental or neurological disorders. This is of great significance for understanding the pathology of various diseases related to dysfunction of 5-HTDRN-BNST neurotransmission and for developing new approaches that provide a theory and basis for more effective therapeutic drugs or treatment methods.

## Author contributions

XZ: Writing – original draft. DL: Writing – original draft. XGY: Writing – original draft. XQY: Writing – original draft, Writing – review & editing. YW: Writing – original draft, Writing – review & editing.
